# Study on Curing Kinetics and the Mechanism of Ultrasonic Curing of an Epoxy Adhesive

**DOI:** 10.3390/polym14030512

**Published:** 2022-01-27

**Authors:** Zhaoyi Liu, Hui Wang, Yizhe Chen, Guodong Kang, Lin Hua, Jindong Feng

**Affiliations:** 1 Hubei Key Laboratory of Advanced Technology for Automotive Components, Wuhan University of Technology, Wuhan 430070, China; markliu3@163.com; 2 Hubei Collaborative Innovation Center for Automotive Components Technology, Wuhan 430070, China; kgd@whut.edu.cn (G.K.); fengjd1996@163.com (J.F.); 3 Hubei Research Center for New Energy & Intelligent Connected Vehicle, Wuhan University of Technology, Wuhan 430070, China

**Keywords:** epoxy adhesive, ultrasonic curing, curing kinetics, accelerated curing mechanism, non-thermal effect, antlion optimization (ALO) algorithm

## Abstract

Ultrasonic curing is an effective way to enhance the curing extent of composite material bonding in the aerospace industry. The non-thermal effect of ultrasonic has been revealed to improve curing efficiency. However, the mechanism of the ultrasonic non-thermal effect is still not clear. In this work, a variable activation energy model of ultrasonic curing was established by utilizing the iso-conversional method, including the activation energy of the thermal effect and activation energy of the non-thermal effect. The thermal effect caused by ultrasonic was accurately peeled off. An obvious decrease in activation energy was found from 54 kJ/mol in thermal curing to 38 kJ/mol in ultrasonic curing. The activation energy of the reaction system in ultrasonic curing was substituted into the modified Kamal autocatalytic equation, and the parameters of the ultrasonic curing kinetic model were estimated by means of an ALO algorithm. Further discussion based on in situ FTIR showed that the non-thermal effect of ultrasonic can affect the vibration strength, stability, and chemical bond energy of internal groups, but cannot cause the fracture of chemical bonds. Moreover, frontier molecular orbital analysis showed that the chemical reactivity of epoxy/amine molecules increased and the HOMO–LUMO energy gap decreased from 6.511 eV to 5.617 eV under the effect of ultrasonic.

## 1. Introduction

Epoxy adhesive is widely used in aerospace [1], vehicle transportation [2], construction [3], electronics [4] and other industries because of its good characteristics (mechanical strength, corrosion resistance, chemical stability, and heat resistance) as well as good adhesion to various substrates. The curing process of epoxy adhesive is the key procedure affecting the properties of cured products [5]. As a common field auxiliary method, ultrasonic is used in the epoxy curing process to improve energy utilization and reduce curing time as well as manufacturing costs [6,7].

The huge amount of energy generated by ultrasonic cavitation destroys the connection through the physical interaction between molecules, thus accelerating the movement of the molecules. These effects reduce the viscosity, promote the crosslinking reaction, and accelerate the curing. Sharma et al. [6] reduced the complete curing time of the resin from 20 min to 5 min. Wang et al. [8] developed an in situ ultrasonic curing process, where the curing rate was 25 times that of thermal curing, but ultrasonic did not change the curing reaction process, which still conformed to the Kamal curing kinetic model. In addition, under the action of ultrasonic, the molecular units can be recombined to form a more uniform and dense crosslinking structure so as to improve the mechanical properties of the cured products.

Differential scanning calorimetry (DSC) has been widely used to determine the glass transition temperature (T_g_) and curing kinetics of thermosetting materials. T_g_ is one of the key parameters that determine the final properties of cured polymers. Hardis [9] and Kim [10] established a nonlinear relationship between T_g_ and the curing degree (α) of epoxy resin by using a Di-Benedetto equation. The curing degree trends obtained from DSC, Raman spectroscopy, and dielectric analysis (DEA) were in good agreement, which provided an idea for in situ curing monitoring in the manufacturing process. Barrett, Borchardt–Daniels, Kissinger, Friedman, and Ozawa–Flynn–Wall methods are commonly used to calculate parameters of dynamic models. In recent years, intelligent algorithms have been widely used to solve the parameters of phenomenological curing kinetic equations. Pagano [11,12] and Hsiao [13] utilized particle swarm optimization (PSO) and a genetic algorithm (GA) as optimizers to estimate the parameters of the curing kinetics model and improve the accuracy of the model. Pagano [11] used the hybrid neural network model to describe the curing dynamics, combined with the PSO method to design the curing system in the filament winding process, which increased the curing degree and shortened the processing time.

In the epoxy/amine reaction system, the rate of the reaction depends on the nucleophilicity of the incoming nucleophile and the leaving capacity of the leaving group which is replaced or substituted [14]. At present, many studies are being carried out on the regioselectivity of nucleophilic addition to substitution, which was predicted using Fukui’s frontier molecular orbital contribution analysis [15,16]. However, previous studies have not explained the change trend of activation energy under ultrasonic accelerated curing and the non-thermal effect of the ultrasonic on epoxy/amine curing mechanism. In this paper, the curing behavior of commercial epoxy structural adhesive 3M DP420 under thermal and ultrasonic curing processes was studied by non-isothermal DSC. The corresponding curing kinetic models were established by using an antlion optimization (ALO) algorithm, and the mechanism of ultrasonic accelerated curing was revealed by using in situ Fourier transform infrared spectroscopy (FTIR) and frontier molecular orbital analysis with software Gaussian 09W.

## 2. Materials and Methods

### 2.1. Materials

A bicomponent adhesive Scotch-Weld^TM^ DP420 Off-White provided by 3M Corporation was used in this study. Component A of the adhesive mainly contains bisphenol A epoxy resin while component B mainly contains diethylene glycol bis (3-aminopropyl) ether, 2,4,6-tris (dimethylaminomethyl) phenol, and polyacrylic acid. The mix ratio of components A and B was 2:1 by volume. At room temperature, the viscosity of the adhesive was 4.5 Pas, the worklife was 20 min, and the curing time was 24 h.

### 2.2. Specimen Preparation

An ultrasonic curing experimental platform was built on the ultrasonic vibration device (MAXWIDE^®^ ME-1800) provided by MingHe Co., Ltd. (Taiwan, China). This platform had a rated power of 1800 W and rated output frequency of 20 kHz. To avoid excessive temperature rise, a pulsed ultrasonic mode was used, which was applied in a cycle of 4 s load/2 s pause. The experimental group and control group were the resin samples treated with and without ultrasonic, respectively. After processing for different time, samples were taken for DSC and FTIR tests. The samples at the processing time of 0 s were labeled as the control group and were used for the thermal curing analysis. A thermocouple thermometer EL-R19, which was provided by EnLai Automation Technology Co., Ltd. (Xiamen, China), was used to monitor the internal temperature of the adhesive during ultrasonic curing. Thermocouple wire (model number: TT-K-24) was provided by OMEGA Engineering Inc. (Norwalk, CT, USA).

### 2.3. Differential Scanning Calorimetry (DSC)

The change in heat flow with time or temperature was recorded by a differential scanning calorimeter (DSC 214 polyma, produced by Netzsch Instrument Manufacturing Co., Ltd., Selb, Germany). DSC samples were prepared at room temperature. A total of 5–7 mg of adhesive sample powder was sealed in an aluminum crucible with a tablet press. A sealed empty crucible was used as a reference. The purge (protective) gas used in the experiment was nitrogen. The gas purity was greater than 99.9%, and the carrier gas flow was about 40 mL/min. The heating rates used in the non-isothermal DSC tests were 5, 10, 15, and 20 K/min.

### 2.4. Fourier Transform Infrared Spectroscopy (FTIR)

The effect of ultrasonic vibration on functional groups was analyzed by in situ FTIR. The equipment used for FTIR testing was the intelligent Fourier infrared spectrometer provided by the Thermo Nicolet Corporation of the United States, with the model of nexus 6700. The FTIR sample preparation process was as follows: 1–2 mg of cured adhesive sample was ground into fine powder and then mixed with 100 mg of dry potassium bromide (KBr) powder. A total of 10 mg of the mixture was taken out and pressed in the die for 1 min. The final tablet thickness was about 0.5–1 mm. The FTIR analysis was carried out in the mid-infrared range from 4000 to 400 cm^−1^ with a resolution of 4 cm^−1^ at room temperature. The FTIR system was located in atmospheric air, and silica gel was used to remove moisture in the air. FTIR spectra of 64 scans were coadded and averaged to obtain the sample spectra. All spectra were performed with a blank KBr pellet as the background and were ratioed against the background.

### 2.5. Antlion Optimization (ALO) Algorithm

**Figure 1:**
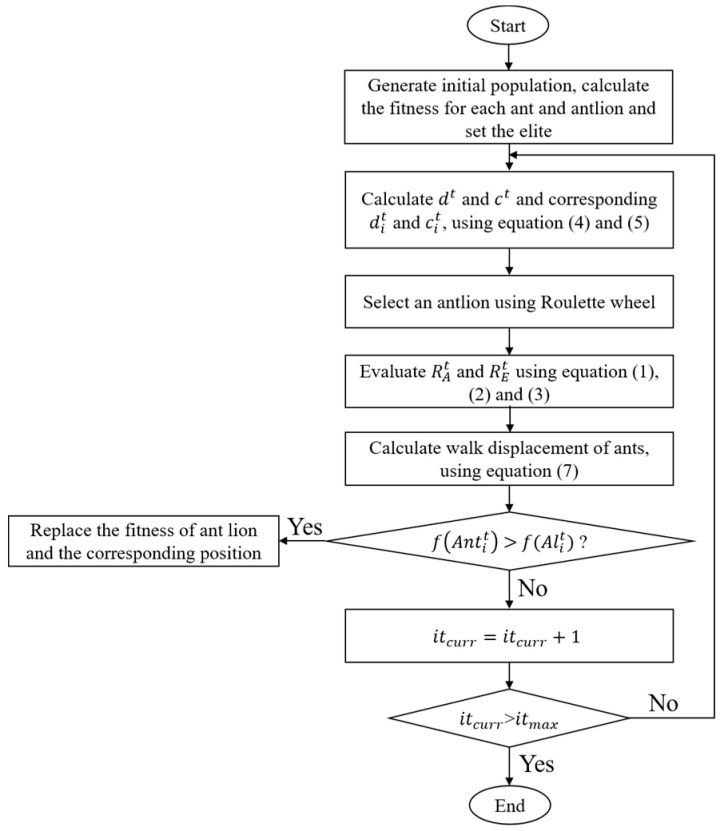
Flow chart of the antlion optimization algorithm.

The ALO algorithm is a new nature-inspired algorithm that was first proposed by Mirjalili in 2015. Its core idea is to simulate the hunting mechanism of antlions hunting ants to achieve global optimization [17]. The process is shown in Figure 1. The Matlab codes for the overall framework of the ALO algorithm are provided in Appendix A. Ants walk randomly in a space to find food. The antlion hunts ants in the sand dune using a designed conical trap. When the randomly walking ants fall into the trap, the antlion catches them and digs the trap again to wait for others.

The process of ants randomly walking around in nature looking for food can be regarded as the process of search agents searching for feasible regions. The process of a random walk can be mathematically expressed as:

(1) $$X\left(t\right)=\left[0,cussum\left(2r\left(t_{1}\right)-1\right),cussum\left(2r\left(t_{2}\right)-1\right),\ldots ,cussum\left(2r\left(t_{n}\right)-1\right)\right]$$

where *X*(*t*) is the set of ants’ randomly walking steps; *cussum* is the cumulative sum of the calculation; *n* is the maximum number of iterations; and *r*(*t*) is a random function, defined as:

(2) $$r\left(t\right)=\{\begin{array}{c} 1,\;\mathrm{if}\;\mathrm{rand}>0.5\\ 0,\;\mathrm{if}\;\mathrm{rand}\leq 0.5 \end{array}$$

where rand is a random number of [0, 1]. Due to a boundary in the feasible region, the position of the ants cannot be updated directly by Equation (1). In order to ensure that ants walk randomly within the feasible range, they need to be normalized according to Equation (3).

(3) $$R_{i}^{t}=\frac{\left(X_{i}^{t}-a_{i}\right)*\left(d_{i}^{t}-c_{i}^{t}\right)}{\left(b_{i}-a_{i}\right)}+c_{i}$$

where $X_{i}^{t}$ is the random displacement of the *i*-th ant in the *t*-th iteration. $R_{i}^{t}$ is the normalized displacement. *a_i_* and *b_i_* are the maximum and minimum displacement of the *i*-th ant, respectively. $c_{i}^{t}$ and $d_{i}^{t}$ define the minimum and maximum displacements of the *i*-th ant in the *t*-th iteration, respectively.

The trap made by the antlion will affect the route of the ant random walk. The following assumptions are put forward:

(4) $$\{\begin{array}{c} c_{i}^{t}=Al_{i}^{t}+c^{t}\\ d_{i}^{t}=Al_{j}^{t}+d^{t} \end{array}$$

where *c^t^*, *d^t^* are the minimum and maximum values of all variables in the *t*-th iteration, respectively. $Al_{j}^{t}$ is the position of the selected *j*-th antlion in the *t*-th iteration. When the ant is trapped in the pit, the antlion will sprinkle sand to prevent it from escaping and then slide down to the center of the pit. In this process, the random walking range of the ants will be sharply reduced. This phenomenon is simulated by the following equation:

(5) $$c^{t}=\frac{c^{t}}{I},\;d^{t}=\frac{d^{t}}{I}$$

(6) $$I=\{\begin{array}{c} 1,\;t\leq 0.1T\\ 10^{\omega }*\frac{t}{T},t>0.1T \end{array}$$

where *I* is the scale coefficient; *T* is the maximum iteration coefficient; *ω* is a number that changes as the number of iterations increases. The ant position updated equation is:

(7) $$Ant_{i}^{t}=\frac{R_{A}^{t}\left(l\right)+R_{E}^{t}\left(l\right)}{2}$$

where $Ant_{i}^{t}$ is the position of the *i*-th ant in the *t*-th iteration. $R_{A}^{t}\left(l\right)$ is the value generated by the ant randomly walking around an antlion selected by a roulette in the *t*-th iteration. $R_{E}^{t}\left(l\right)$ is the value generated in the l-th step of ant random walking around the elite antlion of the *t*-th generation. l is a random value within the number of ant random walk steps.

When the fitness value of the ant is smaller than that of the antlion, it is considered that the antlion can capture it. At this time, the antlion will update the position according to the position of the ant:

(8) $$Al_{j}^{t}=Ant_{i}^{t},\;\;\;\;\;\;if\;f\left(Ant_{i}^{t}\right)>f\left(Al_{j}^{t}\right)$$

## 3. Results and Discussion

### 3.1. Analysis of Adhesive Curing Process

#### 3.1.1. Thermal Curing

**Figure 2:**
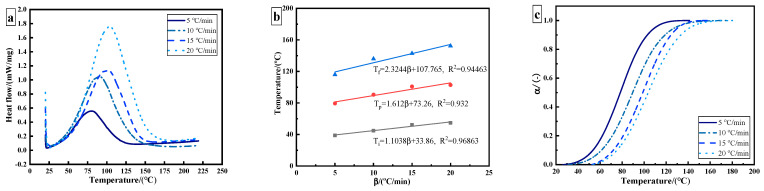
DSC results of 3M DP420 epoxy adhesive: (**a**) dynamic DSC curves of epoxy adhesive at different heating rates; (**b**) characteristic curing temperature; (**c**) curing degree as a function of temperature.

The epoxy adhesive without ultrasonic treatment was tested in thermal curing analysis. Figure 2a shows the dynamic DSC curves of epoxy adhesive under different heating rates. Due to the crosslinking reaction of the adhesive, curing is an exothermic process, and only one exothermic peak appears in the curing process. With the increase in heating rate, the exothermic peak temperature and enthalpy shift towards a high temperature, which is caused by the thermal effect. The characteristic parameters can be obtained, including onset temperature *T_i_*, peak temperature *T_p_*, terminal temperature *T_f_*, and total exothermic reaction heat ΔH, as shown in Table 1. The theoretical curing temperature is obtained by utilizing the T−β extrapolation method. The theoretical curing temperatures of *T_i_*, *T_p_*, and *T_f_* for the used adhesive are 33.86 °C, 73.26 °C, and 107.765 °C, respectively (Figure 2b). The α−T curves are obtained as shown in Figure 2c.

The curing reaction of epoxy resin is a slow–fast–slow process. With the increase in heating rate, the temperature of the maximum curing reaction rate shifts to the right. The temperature corresponding to the maximum curing rate increases from 80 °C to 104 °C. Due to the existence of an induction period, the initial curing rate of an epoxy resin system is relatively slow. With the progression of the reaction, the heat released by the system will accelerate the curing reaction at the same time. In the later stages of the curing reaction, the degree of crosslinking increases, so the curing rate of the system gradually flattens. The fastest reaction rate occurs at the curing degree of 40–60%. The results show that the epoxy adhesive conforms to the autocatalytic curing reaction.

#### 3.1.2. Ultrasonic Curing

**Figure 3:**
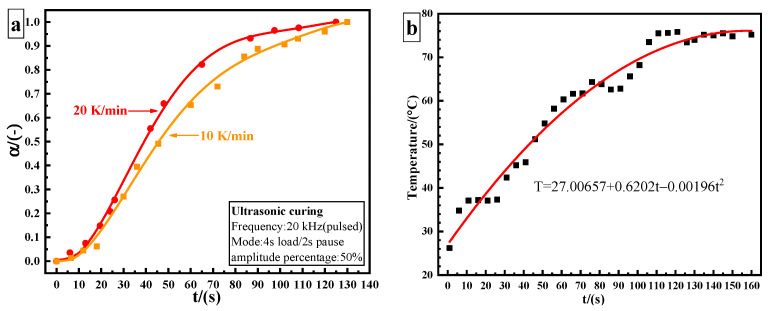
Ultrasonic curing results of 3M DP420 epoxy adhesive: (**a**) curing degree as a function of time; (**b**) internal temperature curve of the adhesive by utilizing ultrasonic curing.

The epoxy adhesive used in ultrasonic curing analysis was treated with ultrasonic vibration and was tested at different processing time until the adhesive was fully cured. Figure 3a shows the curing degree–time fitting curve of ultrasonic curing. The conversion degree α is calculated using Equation (9):

(9) $$\alpha \left(t\right)=1-\frac{\Delta H\left(t\right)}{\Delta H_{total}}$$

where ΔH(t) represents residual heat (J/g) and $\Delta H_{total}$ represents the total heat for the uncured sample (J/g). The total time of ultrasonic curing is 130 s. The conversion curves of ultrasonic and thermal curing show the same trend, which is in agreement with the characteristics of autocatalytic reaction. The obvious difference lies in the initial stage. The induction period is shortened, and the curing rate significantly increases by utilizing ultrasonic curing. However, the curing rate still shows a slow–fast–slow trend. In the ultrasonic curing process, the temperature–time relationship can be described by a quadratic polynomial. The final temperature stabilizes at about 75 °C, as shown in Figure 3b.

### 3.2. Curing Kinetic Analysis

For the curing process, the reaction rate can be expressed by the following formula, and k(T) is generally assumed to follow the Arrhenius equation:

(10) $$\frac{d\alpha }{dt}=k\left(T\right)f\left(\alpha \right)=Aexp\left(-\frac{E_{a}}{RT}\right)f\left(\alpha \right)$$

where, α is the conversion rate, t is the time (min), k(T) is the temperature relationship of the rate constant (min^−1^), A is the pre-exponential factor (min^−1^), $E_{a}$ is the activation energy (kJ/mol), and R is the universal gas constant, and its value is 8.314 J/(mol·K). *T* is the thermodynamic temperature, f(α) is the reaction mechanism function. For thermosetting materials, the Kamal autocatalytic model is commonly used to describe the curing process, and its expression is:

(11) $$f\left(\alpha \right)=\left(k_{1}+k_{2}\alpha^{m}\right){\left(1-\alpha \right)}^{n}$$

#### 3.2.1. Estimation of Apparent Activation Energy

**Figure 4:**
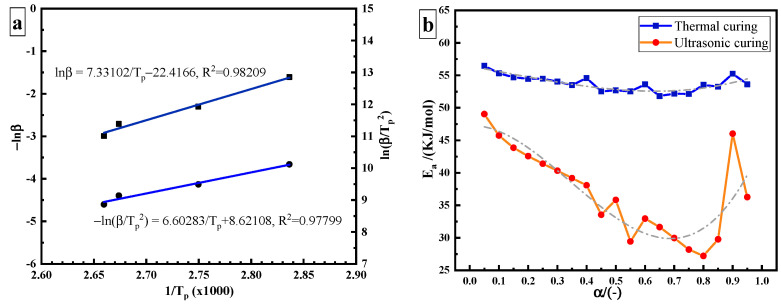
Estimation of the apparent activation energy: (**a**) constant *E_a_*: Kissinger method and Ozawa method; (**b**) variable *E_a_*: FRL method.

In the study of curing kinetics, one of the most important parameters is the apparent activation energy $E_{a}$. At present, two methods are mainly applied to calculate the constant $E_{a}$ of the curing reaction by non-isothermal methods: the Ozawa method and the Kissinger method [18]. The Ozawa method formula is as follows:

(12) $$\frac{d\left(-\ln\beta \right)}{d\left(1/T_{p}\right)}=\frac{1.052E_{a}}{R}$$

where β is the heating rate (K/min) and $T_{p}$ is the peak temperature (K). The values of −lnβ and $1/T_{p}$ can be calculated where −lnβ and $1/T_{p}$ are taken as the abscissa and the ordinate, respectively, as shown in Figure 4a. The $E_{a}$ calculated from its slope (7.33102) is 57.937 kJ/mol. The Kissinger method formula is as follows:

(13) $$\ln\left(\frac{\beta }{T_{p}^{2}}\right)=\ln\left(\frac{AR}{E_{a}}\right)-\frac{E_{a}}{RT_{p}}$$

From the data in Table 1, the value of $\ln\left(\beta /T_{p}^{2}\right)$ can be obtained where −$1/T_{p}$ and $\ln\left(\beta /T_{p}^{2}\right)$ are taken as the abscissa and the ordinate, respectively, as shown in Figure 4a. The $E_{a}$ calculated from its slope (6.60283) is 54.895 kJ/mol. The variable activation energy is calculated using the Friedman–Reich–Levi (FRL) iso-conversional method. The equation is:

(14) $$\ln\left[\frac{\beta d\alpha }{dT}\right]=\ln Af\left(\alpha \right)-\frac{E_{a}}{RT}$$

The blue curve in Figure 4b is the curve of the $E_{a}$ of thermal curing as a function of curing degree. During thermal curing, it can be seen that $E_{a}$ is highest at the beginning of the reaction, and then it decreases with the increase in curing degree. Amine is an effective hydrogen bond-providing molecule, which promotes the ring opening of the epoxy group in the same way as the hydroxyl group of epoxy resin, resulting in the automatic acceleration effect of the curing reaction and the reduction of $E_{a}$. Due to the low hydroxyl content in the initial stage of the reaction, the catalytic effect is not obvious. With the progression of the reaction, the content of hydroxyl increases, which effectively promotes the catalytic reaction and reduces $E_{a}$. However, $E_{a}$ varies in a narrow range from 51.8 kJ/mol to 56.5 kJ/mol, with a variation range of about 5 kJ/mol. The average value is 53.72 kJ/mol. This result is similar to that obtained by the Ozawa (57.937 kJ/mol), and Kissinger methods (54.895 kJ/mol).

The red curve in Figure 4b is the $E_{a}$ curve of ultrasonic curing as a function of α. It can be seen that the range of $E_{a}$ in ultrasonic curing becomes wider, from 27 kJ/mol to 49 kJ/mol, and the value of variation is greater than 20 kJ/mol. Compared with the thermal curing based on heat conduction and heat diffusion, the non-thermal effect caused by ultrasonic may increase the pre-exponential factor or reduce the $E_{a}$ required to initiate a curing reaction, so as to accelerate the epoxy/amine reaction [19]. It is speculated that ultrasonic plays a “catalytic” role in the curing process. $E_{a}$ required for ultrasonic curing is much lower than that required for thermal curing. The energy barrier of the reaction is reduced.

**Figure 5:**
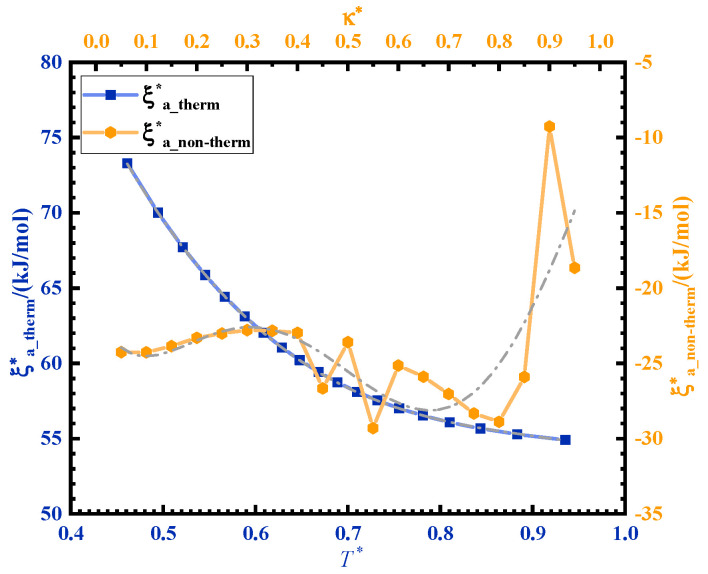
Variation in the activation energy corresponding to thermal and non-thermal effects in ultrasonic curing.

The activation energy of ultrasonic curing $E_{a\_US}$ is divided into two parts: the non-thermal effect activation energy $\xi_{a\_therm}^{*}$ and thermal effect activation energy $\xi_{a_{non}-therm}^{*}$. Ultrasonic curing can be regarded as a non-isothermal curing process. A $\xi_{a\_therm}^{*}-T^{*}$ curve can be obtained from the $E_{a\_therm}-T$ curve so as to obtain the contribution of the non-thermal effect of ultrasonic in the curing process. $T^{*}$ is the normalization of T. The time ratio of the ultrasonic curing κ is as an independent variable, κ∈[0,t/f]. t is the total time of ultrasonic curing and f is the ultrasonic cycle. *κ* is normalized as $\kappa^{*}$. The fitting curve between $\kappa^{*}$ and $\xi_{a{\_}_{non}-therm}^{*}$ is shown in Figure 5. The activation energy of ultrasonic curing $E_{a\_US}$ can be expressed by the following formula:

$$\begin{array}{c} E_{a_{\_US}}=\xi_{a\_therm}^{*}+\xi_{a_{non}-therm}^{*}\\ \xi_{a\_therm}^{*}=181.545-399.051*T^{*}+428.244*{\left(T^{*}\right)}^{2}-156.516*{\left(T^{*}\right)}^{3}\\ \xi_{a_{non}-therm}^{*}=-21.646-69.792*\kappa^{*}-69.792*{\left(\kappa^{*}\right)}^{2}+548.102*{\left(\kappa^{*}\right)}^{3}+1688.992*{\left(\kappa^{*}\right)}^{4}-618.487*{\left(\kappa^{*}\right)}^{5} \end{array}$$

$\xi_{a\_therm}^{*}$ decreases with temperature rise during the curing process. The temperature rise is caused by ultrasonic high-frequency vibration and friction. $\xi_{a{\_}_{non}-therm}^{*}$ is the contribution of ultrasonic energy, which is negative and varies from −29.3 kJ/mol to −9.3 kJ/mol.

#### 3.2.2. Curing Kinetic Model

##### Kinetic Model of Thermal Curing

The average activation energy (56.416 kJ/mol) calculated by the Ozawa and Kissinger methods is substituted into Formula (10) to calculate the parameters of the kinetic model. Using the Levenberg–Marquardt iterative algorithm in software Origin Pro 2018, nonlinear fitting is utilized to determine the kinetic model parameters of thermal curing. The Kamal autocatalytic model parameters of thermal curing are shown in Table 2.

The modified Sun–Gang model was used to describe the Kamal autocatalytic curing kinetics model with variable $E_{a}$. $E_{a}$ and lnA were assumed to be a function of α. The equation can be expressed as:

(15) $$\frac{d\alpha }{dt}=e^{\ln\left[A\left(\alpha \right)f\left(\alpha \right)\right]}e^{\left[-E\left(\alpha \right)/RT\right]}$$

where $\ln A=\varphi_{1}+\varphi_{2}\alpha +\varphi_{3}\alpha^{2}+\varphi_{4}\alpha^{3}$; $E\left(\alpha \right)=\varphi_{5}+\varphi_{6}\alpha +\varphi_{7}\alpha^{2}+\varphi_{8}\alpha^{3}$. The values of $\varphi_{5}$, $\varphi_{6}$, $\varphi_{7}$, and $\varphi_{8}$ can be obtained by polynomial fitting, as listed in Table 3. The ALO algorithm is used as an optimization tool to determine the parameters of the model. The least square method is used as a fitness function to evaluate the target value, as shown in Formula (16). The operation of ALO is carried out on MATLAB R2019b. The operating environment is a 64-bit Win10 system (Intel (R) core (TM) i7-10870H, 2.60 Hz; RAM 32 GB). The population size is set to 25 and the maximum number of iterations is set to 15,000. The ranges of the fitting parameters are shown in Table 2 and Table 4.

(16) $$F\left(x\right)=\frac{\sum_{i=1}^{n}{\left(f\left(x_{i}\right)-y_{i}\right)}^{2}}{n}$$

**Figure 6:**
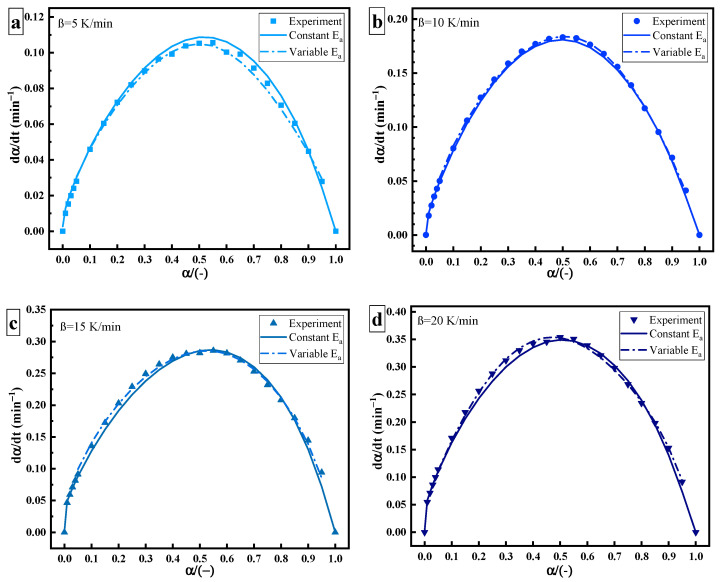
Curing rate–curing degree curves of thermal curing: heating rate of (**a**) 5 K/min; (**b**) 10 K/min; (**c**) 15 K/min; (**d**) 20 K/min.

Figure 6 shows the relationship between dα/dt and α, including experimental data (point), data of constant $E_{a}$ (solid line), and data of variable $E_{a}$ (dotted line). The derivation of variable $E_{a}$ is more in line with the actual process of the curing reaction. As the curing reaction proceeds, the curing system changes from gelation (liquid to rubber) and vitrification (rubber to glass). The crosslinking reaction occurs easier, the fluidity of molecules decreases, the free volume becomes smaller, and the curing changes from a kinetic state to a diffusion state. Therefore, there is a slight deviation between the data of constant $E_{a}$ and the experimental data at a high solidifying degree. This may be related to the change in the diffusion effect or reaction mechanism [20,21].

##### Kinetic Model of Ultrasonic Curing

**Figure 7:**
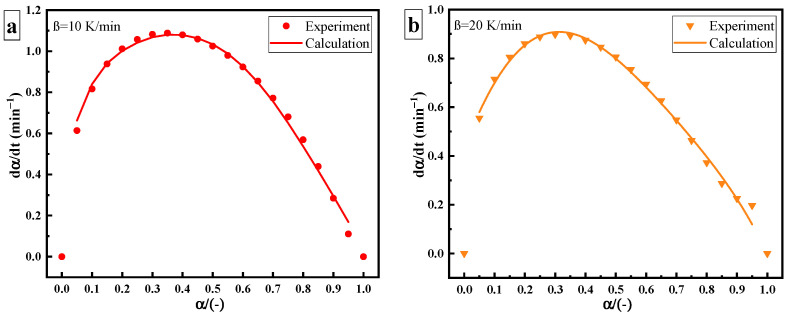
Curing rate versus curing degree of ultrasonic curing: heating rate of (**a**) 10 K/min; (**b**) 20 K/min.

Compared with thermal curing, the rate of ultrasonic curing significantly increases, as shown in Figure 7. The deviation between the thermal and ultrasonic curing rate curves may be caused by the change in the curing mechanism. Under ultrasonic “catalysis”, the maximum curing rate of the epoxy amine system shifts to the left at a curing degree of 40%, but its overall trend (slow–fast–slow) remains unchanged. The Kamal autocatalytic model can still be used to describe the curing reaction process. Since $E_{a}$ changes greatly under ultrasonic, it needs to be described by the variable activation energy autocatalytic reaction model. The model parameters are as shown in Table 4.

### 3.3. Glass Transition Analysis

The glass transition temperature is not only a measure of the adhesive service temperature, but also an index of the matrix curing state [22]. The Di-Benedetto equation is used to describe the nonlinear relationship between T_g_ and α. The formula is:

(17) $$\frac{T_{g}-T_{g0}}{T_{g\infty }-T_{g0}}=\frac{\lambda \alpha }{1-\left(1-\lambda \right)\alpha }$$

**Figure 8:**
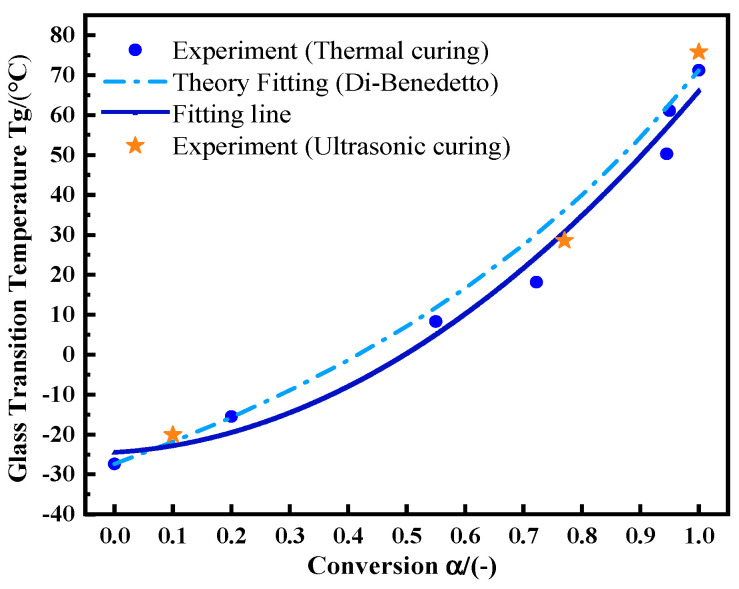
The relationship between T_g_ and α by utilizing the Di-Benedetto equation.

T_g0_ is the T_g_ value of the uncured sample, and T_g__∞_ is the maximum T_g_ value of the fully cured sample. *λ* is a parameter related to the structure, which is theoretically equal to the step change in the heat capacity at T_g_ and T_g0_, $\Delta C_{p\infty }/\Delta C_{p0}$. $\Delta C_{p0}$ is the change in the specific heat capacity of the uncured adhesive during glass transition. $\Delta C_{p\infty }$ is the change in the specific heat capacity during the glass transition of the fully cured adhesive. First, samples were kept at 75 °C for 0, 5, 15, 30, 45, 50, and 60 min, respectively. Then, the temperature was dropped rapidly to −50 °C. After that, the temperature was raised to 180 °C at 10 °C/min. The T_g_ values and reaction residual heat values were recorded, from which α was calculated. *λ* was calculated from Formula (17). Its value is 0.537. Figure 8 shows the experimental data and fitting curve of thermal curing. With the increase in curing degree, T_g_-α shows more obvious nonlinear behavior [23]. After ultrasonic curing for 20 s, 60 s, and 130 s, the adhesive was sampled for the non-isothermal DSC test under the same conditions as the thermal curing samples. Ultrasonic curing still meets the same change law as thermal curing (orange asterisk). A slightly higher T_g_ value of 75.76 °C is achieved by ultrasonic curing.

### 3.4. Mechanism Analysis of Ultrasonic Accelerated Curing

**Figure 9:**
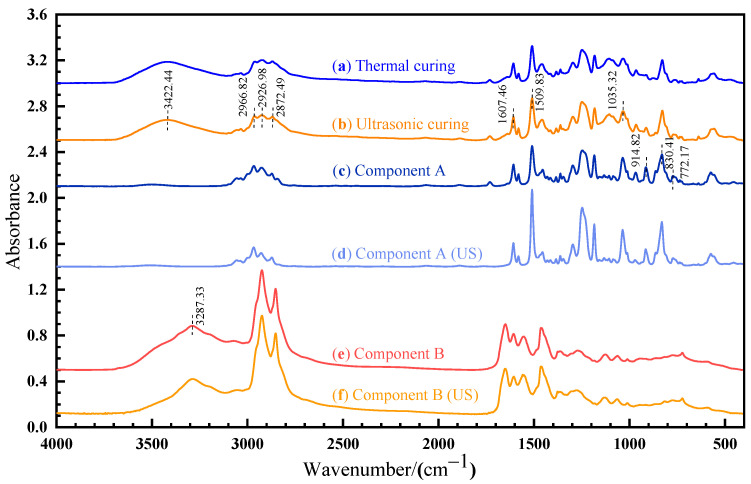
FTIR spectrum of: (**a**) thermal cured sample; (**b**) ultrasonic cured sample; (**c**) component A; (**d**) ultrasonic-treated component A; (**e**) component B; (**f**) ultrasonic-treated component B.

The OMNIC software (version 9.2) was used to analyze the FTIR results, as shown in Figure 9. A sharp absorption peak at 830.41 cm^−1^ and 914.82cm^−1^ corresponds to the stretching C–O–C of the oxirane group and the vibration of C–O in the epoxy group, respectively. The peak observed at 3287.33 cm^−1^ is caused by the -NH_2_ vibrational absorption of amine in component B. For the cured sample, a new band appears at 3422.44 cm^−1^, which is attributed to the vibration of the O–H group [24]. As shown in Figure 9a,b, the spectra recorded for thermal and ultrasonic cured samples are identical, suggesting that the chemical mechanism is the same for thermal and ultrasonic curing.

According to ISO 20368, the peak at 1607.46 cm^−1^ (stretching C=C of the aromatic ring) and 2872.49 cm^−1^ (stretching C–H) can be used as a benchmark for calculating the characteristic absorption peak. After ultrasonic vibration, the absorption peak ratio of the C–O bond (914.82 cm^−1^) decreases from 0.873 to 0.753 and the absorption peak ratio of the C–O–C bond (830.41 cm^−1^) increases from 1.220 to 1.806, as shown in Figure 9c,d. The absorption peak ratio of the -NH_2_ bond (3287.33 cm^−1^) decreases from 0.896 to 0.837, as shown in Figure 9e,f. Ultrasonic vibration changes the molecular configuration. The rearrangement of the electron cloud causes the change in the chemical bond vibration intensity and dipole moment, which affects the corresponding absorption peak intensity and width. The non-thermal effect of ultrasonic can affect the vibration strength, stability, and chemical bond energy of internal groups, but cannot cause the fracture of chemical bonds.

In order to evaluate the effect of changes in molecular structure under ultrasonic on the chemical reactivity of the epoxy/amine system, density functional theory (DFT) was conducted at the B3LYP/6-311G(d,p) level. Using the keyword “field” in UFF mode, the ultrasonic field is equivalent to the applied force field on molecules. Under the framework of the Koopmans approximation, the parameters related to the global reactivity descriptors are determined, including the energy of the lowest unoccupied molecular orbital (ELUMO), the energy of the highest occupied molecular orbital (EHOMO), electronegativity (χ), global hardness (η), and the global electrophilicity index (ω) [25]. The global electrophilicity index (ω) characterizes the electrophilicity of the molecule. Parr defined ω as in ref. [26]

(18) $$\omega =\frac{\chi^{2}}{2\eta }\;$$

Global hardness (*η*) has been defined as $\varepsilon_{LUMO}-\varepsilon_{HOMO}$. Electronegativity (*χ*) has been defined as $-\left(\varepsilon_{HOMO}+\varepsilon_{LUMO}\right)/2$. Global reactivity indices are shown in Table 5.

**Figure 10:**
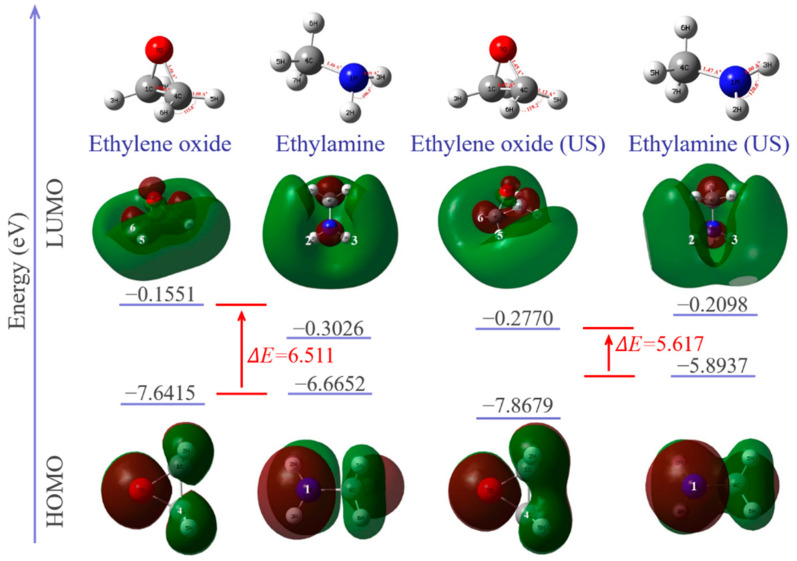
Optimized geometry of each molecule and HOMO/LUMO orbitals with energy levels.

Calculated HOMO, LUMO and optimized geometries along with energy diagrams are summarized in Figure 10. The electron densities of the HOMO orbitals of methylamine are mainly distributed around N–H and centered around the nitrogen atom, indicating that the center of the nucleophilic reaction is there. Ultrasonic vibration increases the electron density around the nitrogen atom in HOMO, which represents electron donors, and its energy is associated with the ionization potential. The increase in HOMO value (from −0.24494 A.U. to −0.21659 A.U.) and decrease in the electrophilicity index (from 0.95381 eV to 0.81926 eV) of the methyl amine molecule indicates that electrons are easier to transition from the low-energy HOMO orbit to the high-energy LUMO orbit. The nucleophilicity of methyl amine is thus enhanced under ultrasonic vibration. The LUMO orbitals of ethylene oxide are mainly around the C–H of the carbon atoms, indicating that the center of the electrophilic reaction is there. Under ultrasonic vibration, carbon atoms can provide wider empty orbits and accept electrons more easily. The energy of LUMO corresponds to the electron affinity. The increase in the LUMO value (from −0.0057 A.U. to −0.01018 A.U.) and electrophilicity index (from 1.01496 A.U. to 1.09242 A.U.) of the ethylene oxide molecule indicates that its electrophilicity is enhanced under ultrasonic vibration.

The HOMO–LUMO energy gap explains the concluding charge transfer interaction within the molecule and is useful in determining molecular electrical transport properties. A lower frontier orbital gap (HOMO–LUMO energy gap) with a value of 5.617 eV is achieved under ultrasonic vibration. The epoxy/amine system has a higher chemical reactivity and lower kinetic stability. It means that ultrasonic promotes the curing reaction of the epoxy/amine system.

## 4. Conclusions

The curing behavior and ultrasonic accelerated curing mechanism of an epoxy adhesive were studied. The following conclusions can be drawn from this study:

(1) A model of the activation energy in ultrasonic curing was established to describe the contribution of the ultrasonic non-thermal effect. It was found that the non-thermal effect of ultrasonic reduces the energy barrier of the curing reaction. The ALO algorithm was utilized to estimate the curing kinetic parameters, and an extremely good agreement between the experimental and predicted data was achieved.

(2) The nonlinear relationship between the glass transition temperature and the curing degree in ultrasonic curing was established and fitted with a Di-Benedetto equation.

(3) The non-thermal effect of ultrasonic was investigated by using in situ FTIR. The non-thermal effect can affect the vibration strength, stability, and chemical bond energy of the internal groups, but cannot cause the fracture of chemical bonds.

(4) Chemical reactivity and HOMO–LUMO energy levels were obtained utilizing frontier molecular orbitals. The increase in chemical reactivity and decrease in the HOMO–LUMO energy gap explained the promoting effect of ultrasonic on the molecular dynamics.

## Figures and Tables

**Table 1:** Characteristic parameters of curing process at different heating rates.

Heating Rate (°C/min)	*T_i_* (°C)	*T_p_* (°C)	*T_f_* (°C)	Δ*T* (°C)	Δ*H* (J/g)
5	38.86	79.41	115.99	77.13	285.48
10	44.77	90.54	135.97	91.2	328.93
15	52.23	100.85	142.90	90.67	232.74
20	54.77	102.84	152.42	97.65	296.34

**Table 2:** Kamal autocatalytic model parameters of thermal curing.

**Curing Kinetic Model Parameters Obtained with a Constant *E_a_***						
**Parameters**	**Range**	**5 K/min**	**10 K/min**	**15 K/min**	**20 K/min**	**Average Value**
ln *A*	[5, 20]	17.607	17.249	16.957	17.015	17.207
*k*_1_	[0, 5]	0.615	0.407	0.801	0.754	0.644
*k*_2_	[0, 5]	1.058	1.949	1.820	1.930	1.689
*m*	[0, 5]	0.198	0.102	0.115	0.117	0.133
*n*	[0, 5]	1.374	1.506	1.248	1.417	1.386
*R* ^2^	-	0.9773	0.9792	0.9828	0.9861	-
**Curing Kinetic Model Parameters Obtained with a Variable *E_a_***						
$\varphi_{1}$	[−100, 100]	16.932	17.510	16.833	17.012	17.073
$\varphi_{2}$	[−100, 100]	−3.860	−5.042	−4.060	−2.077	−3.759
$\varphi_{3}$	[−100, 100]	0.781	2.891	0.901	−2.572	0.501
$\varphi_{4}$	[−100, 100]	1.009	0.391	1.893	3.875	1.792
*k*_1_	[0, 5]	1	1.020	1.901	1.245	1.292
*k*_2_	[0, 5]	2	1.861	1.997	1.063	1.730
*m*	[0, 5]	0.118	0.364	0.275	0.102	0.215
*n*	[0, 5]	0.786	1.199	1	1.149	1.034
*R* ^2^	-	0.9862	0.9862	0.9866	0.9870	-

**Table 3:** Parameters for ln *A* and *E*(*α*).

Parameters	Thermal Curing	Ultrasonic Curing
$\varphi_{5}$	56.417	47.275
$\varphi_{6}$	−8.111	1.275
$\varphi_{7}$	−3.139	−114.249
$\varphi_{8}$	10.016	109.958

**Table 4:** Kamal autocatalytic model parameters of ultrasonic curing.

Curing Kinetic Parameters Obtained with a Variable *E_a_*				
Parameters	Range	10 K/min	20 K/min	Average Values
$\varphi_{1}$	[−100, 100]	19.334	17.374	18.354
$\varphi_{2}$	[−100, 100]	−4.494	0.011	−2.241
$\varphi_{3}$	[−100, 100]	−35.397	−42.287	38.842
$\varphi_{4}$	[−100, 100]	36.727	41.457	39.092
k_1_	[0, 5]	0.120	0.567	0.343
k_2_	[0, 5]	1.953	1.967	1.960
m	[0, 5]	0.664	0.432	0.548
n	[0, 5]	1.029	1.482	1.256
R^2^	-	0.9724	0.9735	-

**Table 5:** Global reactivity indices, calculated at the B3LYP/6-311G (d,p) level.

	Ethylene Oxide	Ethylene Oxide (US)	Methyl Amine	Methyl Amine (US)
HOMO (A.U.)	−0.28082	−0.28914	−0.24494	−0.21659
LUMO (A.U.)	−0.0057	−0.01018	−0.01112	−0.00771
Electronegativity *χ* (eV)	3.898303	4.072456	3.48387	3.05175
Chemical hardness *η* (eV)	7.48639	7.59089	6.36257	5.68391
Electron affinity *I* (eV)	−0.1551	−0.27701	−0.30259	−0.20979
Ionization potential *A* (eV)	−7.64150	−7.86789	−6.66516	−5.89371
Electrophilicity index *ω* (eV)	1.01496	1.09242	0.95381	0.81926

## Data Availability

Data are contained within the article.

## Appendix A

### Appendix A.1. Matlab Codes for the Framework of the ALO

The ALO matlab codes used in this paper were developed from the code of S. Mirjalili [17].

*Initialize the first population of ants and antlions randomly*
*Calculate the fitness of ants and antlions*
*Find the best antlions and assume as the elite (determined optimum)*
***while****the end criterion is not satisfied*
*** for****every ant*
* Select an antlion using Roulette wheel*
* Update c and d in Equation (5)*
* Create a random walk and normalize it using Equations (1) and (3)*
* Update the position of ant using Equation (7)*
* **end for***
* Calculate the fitness of all ants*
* Replace an antlion with its corresponding ant it if becomes fitter (Equation (8))*
* Update elite if an antlion becomes fitter than the elite*
***end while***
***Return****elite*

### Appendix A.2. Matlab Codes for the Function A

*%% This function defines the optimization equation and fitness function*
***function*** *y = funobj(X)*
*% Curing kinetic equation*
*ytemp = exp(p1 + p2.*x + p3.*x.^2 + p4.*x.^3).*…* *exp(−1000.*(phi(5) + phi(6).*x + phi(7).*x.^2 + phi(8).*x.^3)./(R*pT)).*(k1 + k2.*x.^m).*(1 − x).^n;*
*% Mathematical formulation of the fitness function*
*F_Curv = ytemp;*
*y = sum((F_Curv-py).^2);*
*y = y/Num;*
***end***

### Appendix A.3. Matlab Codes for the Function B

*%% This function creates the first random population*
***function*** *X = initialization(SearchAgents_no,dim,ub,lb)*
*Boundary_no = size(ub,2); % number of boundaries*
*% If the boundaries of all variables are equal and user enter a signal*
*% number for both ub and lb*
***if*** *Boundary_no == 1*
* X = rand(SearchAgents_no,dim).*(ub − lb) + lb;*
***end***
*% If each variable has a different lb and ub*
***if*** *Boundary_no > 1*
* **for** i = 1:dim*
* ub_i = ub(i);*
* lb_i = lb(i);*
* X(:,i) = rand(SearchAgents_no,1).*(ub_i-lb_i) + lb_i;*
* **end***
***end***

### Appendix A.4. Matlab Codes for the Function C

*%% This function creates random walks*
***function*** *X = initialization(SearchAgents_no,dim,ub,lb)*
***function*** *[RWs] = Random_walk_around_antlion(Dim,max_iter,lb, ub,antlion,current_iter)*
***if*** *size(lb,1) == 1 && size(lb,2) == 1 %Check if the bounds are scalar*
* lb = ones(1,Dim)*lb;*
* ub = ones(1,Dim)*ub;*
***end***
*if size(lb,1) > size(lb,2) %Check if boundary vectors are horizontal or vertical*
* lb = lb’;*
* ub = ub’;*
*end*
*I = 1; % I is the ratio in Eq. (6)*
***if*** *current_iter>max_iter/10*
* I = 1 + 100*(current_iter/max_iter);*
***end***
*……*
***if*** *current_iter > max_iter*(0.95)*
* I = 1 + 1000000*(current_iter/max_iter);*
***end***
*% Decrease boundaries to converge towards antlion*
*% Equation (5) in the paper*
*lb = lb/(I);*
*ub = ub/(I);*
*% Move the interval of [lb ub] around the antlion [lb + anlion ub + antlion]*
*% Equation (4) in the paper*
***if*** *rand < 0.5*
* lb = lb + antlion;*
***end***
***if*** *rand >= 0.5*
* * *ub = ub + antlion;*
***else***
* * *ub = −ub + antlion;*
***end***
***for*** *i = 1:Dim*
* X = [0 cumsum(2*(rand(max_iter,1) > 0.5) − 1)’]; % Equation (1) in the paper*
* X_norm = ((X − a).*(d − c))./(b − a) + c; % Equation (3) in the paper*
***end***

### Appendix A.5. Matlab Codes for the Function D

*%% This function creates Roulette Wheel Selection*
***function*** *choice = RouletteWheelSelection(weights)*
* accumulation = cumsum(weights);*
* p = rand() * accumulation(end);*
* chosen_index = −1;*
* **for** index = 1:length(accumulation)*
* **if** (accumulation(index) > p)*
* chosen_index = index;*
* break;*
* **end***
* **end***
* choice = chosen_index;*

### Appendix A.6. Matlab Codes for the Function E

*%% This function creates ALO algorithm startup*
*SearchAgents_no = 25; % Number of search agents*
*lb = […]; ub = […]; % Upper and lower bounds of fitting parameters*
*dim = N;*
*fobj = @funobj;*
*[Best_score,Best_pos,cg_curve] = ALO(SearchAgents_no,Max_iteration,lb,ub,dim,fobj);*

## References

[1] Wu M., Tong X., Wang H., Hua L., Chen Y. (2020). Effect of Ultrasonic Vibration on Adhesive Bonding of CFRP/Al Alloy Joints Grafted with Silane Coupling Agent. Polymers.

[2] Rehman S., Akram S., Kanellopoulos A., Elmarakbi A., Karagiannidis P.G. (2020). Development of new graphene/epoxy nanocomposites and study of cure kinetics, thermal and mechanical properties. Thermochim. Acta.

[3] Michel M., Ferrier E. (2020). Effect of curing temperature conditions on glass transition temperature values of epoxy polymer used for wet lay-up applications. Constr. Build. Mater..

[4] Bratasyuk N.A., Zuev V.V. (2020). The study of the curing mechanism, kinetic and mechanical performance of polyurethane/epoxy composites using aliphatic and aromatic amines as curing agents. Thermochim. Acta.

[5] Akbari V., Jouyandeh M., Paran S.M., Ganjali M.R., Abdollahi H., Vahabi H., Ahmadi Z., Formela K., Esmaeili A., Mohaddespour A. (2020). Effect of Surface Treatment of Halloysite Nanotubes (HNTs) on the Kinetics of Epoxy Resin Cure with Amines. Polymers.

[6] Sharma S., Luzinov I. (2011). Ultrasonic curing of one-part epoxy system. J. Compos. Mater.-J. Compos. Mater..

[7] Du L., Wang Q. (2010). Experimental study on ultrasonic stress relief for cured SU-8 photoresist layer. Microelectron. Eng..

[8] Wang H., Yuan Y., Chen Y. (2020). Characterization and mechanism of accelerated curing of adhesives by in situ ultrasonic vibration for bonded joints. J. Polym. Eng..

[9] Hardis R., Jessop J.L.P., Peters F.E., Kessler M.R. (2013). Cure kinetics characterization and monitoring of an epoxy resin using DSC, Raman spectroscopy, and DEA. Compos. Part A Appl. Sci. Manuf..

[10] Kim Y.C., Hong S., Sun H., Kim M.G., Choi K., Cho J., Choi H.R., Koo J.C., Moon H., Byun D. (2017). UV-curing kinetics and performance development of in situ curable 3D printing materials. Eur. Polym. J..

[11] Pagano R.L., Calado V.M.A., Bezerra de Souza M., Biscaia E.C. (2014). Proposal of an optimum cure cycle for filament winding process using a hybrid neural network—First principles model. Polym. Compos..

[12] Pagano R.L., Calado V.M.A., Tavares F.W., Biscaia E.C. (2008). Cure kinetic parameter estimation of thermosetting resins with isothermal data by using particle swarm optimization. Eur. Polym. J..

[13] Hsiao K.-T., Little R., Restrepo O., Minaie B. (2006). A study of direct cure kinetics characterization during liquid composite molding. Compos. Part A Appl. Sci. Manuf..

[14] Okumoto S.Y.S. (2002). Computational study of epoxy-amine reactions. J. Comput. Chem..

[15] Mirzaei S., Khosravi H. (2017). Predicting the regioselectivity of nucleophilic addition to arynes using frontier molecular orbital contribution analysis. Tetrahedron Lett..

[16] Hajime Hirao T.O. (2003). Theoretical Study of Reactivities in Electrophilic Aromatic Substitution Reactions: Reactive Hybrid Orbital Analysis. J. Phys. Chem. A.

[17] Mirjalili S. (2015). The Ant Lion Optimizer. Adv. Eng. Softw..

[18] Nowruzi Varzeghani H., Amiri Amraei I., Mousavi S.R. (2020). Dynamic Cure Kinetics and Physical-Mechanical Properties of PEG/Nanosilica/Epoxy Composites. Int. J. Polym. Sci..

[19] Wang Y., Luo S., Yang L., Ding Y. (2021). Microwave curing cement-fly ash blended paste. Constr. Build. Mater..

[20] Johnston K., Pavuluri S.K., Leonard M.T., Desmulliez M.P.Y., Arrighi V. (2015). Microwave and thermal curing of an epoxy resin for microelectronic applications. Thermochim. Acta.

[21] Zhou T., Gu M., Jin Y., Wang J. (2005). Studying on the curing kinetics of a DGEBA/EMI-2,4/nano-sized carborundum system with two curing kinetic methods. Polymer.

[22] Xu H., Tian G., Meng Y., Li X., Wu D. (2021). Cure kinetics of a nadic methyl anhydride cured tertiary epoxy mixture. Thermochim. Acta.

[23] Kudisonga C., Villacorta B., Chisholm H., Vandi L.-J., Heitzmann M. (2021). Curing kinetics of a siloxane pre-ceramic prepreg resin. Ceram. Int..

[24] Wang H., Liu Z.Y., Chen Y.Z., Hua L., Qiu Y. (2021). Effect of ultrasonic pretreatment on thermo-mechanical properties of epoxy adhesive. Mater. Res. Express.

[25] Ehouman A., Kouakou A., Diarrassouba F., Ouattara H.A.A., Niamien P.M. (2021). Study of the Stability and Chemical Reactivity of a Series of Tetrazole Pyrimidine Hybrids by the Density Functional Theory Method (DFT). Orient. J. Chem..

[26] Chattaraj P.K., Sarkar U., Roy D.R. (2006). Electrophilicity index. Chem. Rev..

